# Trends in Drug Overdose Mortality Rates in Kentucky, 2019-2020

**DOI:** 10.1001/jamanetworkopen.2021.16391

**Published:** 2021-07-13

**Authors:** Svetla Slavova, Dana Quesinberry, Sarah Hargrove, Peter Rock, Candace Brancato, Patricia R. Freeman, Sharon L. Walsh

**Affiliations:** 1 Kentucky Injury Prevention and Research Center, University of Kentucky, Lexington; 2 Department of Biostatistics, University of Kentucky, Lexington; 3 Department of Health Management and Policy, University of Kentucky, Lexington; 4 Center for Clinical and Translational Science, University of Kentucky, Lexington; 5 Department of Pharmacy Practice and Science, University of Kentucky, Lexington; 6 Department of Behavioral Science, University of Kentucky, Lexington; 7 Center on Drug and Alcohol Research, University of Kentucky, Lexington

## Abstract

This cross-sectional study evaluates the changes in drug overdose mortality rates for Kentucky residents between 2019 and 2020.

## Introduction

In April 2020, early signals from emergency medical services data raised concerns for increasing numbers of drug overdoses during the emerging COVID-19 pandemic in Kentucky.^1^ Subsequently, provisional national data for the 12-month period ending May 2020 showed accelerated drug overdose deaths, resulting in a Health Alert Network Advisory calling for expanded prevention efforts.^2^ This cross-sectional study evaluates the changes in drug overdose mortality rates for Kentucky residents between 2019 and 2020.

## Methods

This study was approved by the University of Kentucky institutional review board and followed the Strengthening the Reporting of Observational Studies in Epidemiology (STROBE) reporting guideline for cross-sectional studies. Informed consent was not required or possible because the study used secondary data for deceased individuals, in accordance with 45 CFR §46.

We used Kentucky Office of Vital Statistics death certificate data (extracted February 8, 2021). Reported 2020 data are provisional, incomplete, and intended as an early warning. Drug overdose deaths (all intents) and drug involvement were identified using *International Statistical Classification of Diseases and Related Health Problems, Tenth Revision*–coded underlying and multiple causes of death^3^ or text search in the cause-of-death section (see the eAppendix in the Supplement). Crude rates^4^ per 100 000 population, 95% CIs, rate ratios (RRs), and *P* values were calculated by demographic group (sex, age group, race/ethnicity, and metropolitan vs nonmetropolitan residency).^5^ Classification by race/ethnicity was based on death certificate information and aimed to identify specific trends in racial/ethnic groups to inform targeted interventions. Statistical tests for significance of 2020 vs 2019 RRs by group were calculated in Poisson regression models with year-group interaction. All *P* values were from 2-sided tests and were considered significant at α = .05. Data were analyzed using SAS statistical software version 9.4 (SAS Institute).

## Results

There were 3225 Kentucky resident overdose deaths in 2019 to 2020; more than 60% were male (2154 residents), 90% were White non-Hispanic (2903 residents), and more than 25% of deaths (923 residents) occurred in the age group 35 to 44 years. There was a 45.06% increase in overdose deaths from 2019 (1316 deaths) to 2020 (1909 deaths). The 2020 crude overdose mortality rate was 42.64 deaths per 100 000 population (95% CI, 40.77-44.59 deaths per 100 000 population), which was significantly higher than that in 2019 (29.46 deaths per 100 000 population; 95% CI, 27.86-31.05 deaths per 100 000 population; *P* < .001) (Table). Kentucky registered a record-high number of deaths in May 2020 (240 deaths) (Figure), a 150% increase from May 2019. Monthly overdose death counts in June to December 2020 remained elevated compared with the same months in any of the previous 9 years.

**Table.** Kentucky Resident Crude Drug Overdose Mortality Rates and Rate Ratios, by Demographic Group, 2019-2020^a^

Demographic characteristics	Deaths, No. (%)		Crude drug overdose mortality rate, deaths/100 000 population (95% CI)		Rate ratio (95% CI)	*P* value
	2019	2020^a^	2019	2020^a^		
All Kentucky residents (n = 3225)	1316	1909	29.46 (27.86-31.05)	42.64 (40.77-44.59)	1.45 (1.35-1.55)^b^	<.001
Sex						
Female (n = 1071)	482 (36.63)	589 (30.85)	21.26 (19.37-23.16)	25.99 (23.89-28.08)	1.22 (1.08-1.38)	.001
Male (n = 2154)	834 (63.37)	1320 (69.15)	37.89 (35.32-40.46)	59.97 (56.74-63.21)	1.58 (1.45-1.73)	<.001
Race/ethnicity						
Black non-Hispanic (n = 270)	105 (7.98)	165 (8.64)	26.47 (21.41-31.53)	41.6 (35.25-47.94)	1.57 (1.23-2.00)	<.001
Hispanic (n = 43)	Suppressed^c^	Suppressed^c^	10.88 (6.55-16.98)	13.74 (8.8-20.44)	1.26 (0.69-2.31)	.45
Other non-Hispanic (n = 9)^d^	≤5	≤5	NA^c^	NA^c^	NA	NA
White non-Hispanic (n = 2903)	1187 (90.20)	1716 (89.89)	31.17 (29.4-32.94)	45.06 (42.93-47.19)	1.45 (1.34-1.56)	<.001
Age group, y						
15-24 (n = 188)	67 (5.09)	121 (6.34)	11.48 (8.89-14.57)	20.73 (17.03-24.42)	1.81 (1.34-2.43)	<.001
25-34 (n = 743)	298 (22.64)	445 (23.31)	50.73 (44.97-56.49)	75.76 (68.72-82.8)	1.49 (1.29-1.73)	<.001
35-44 (n = 923)	366 (27.81)	557 (29.18)	66.43 (59.62-73.24)	101.1 (92.7-109.49)	1.52 (1.33-1.74)	<.001
45-54 (n = 720)	325 (24.70)	395 (20.69)	57.66 (51.39-63.93)	70.08 (63.17-76.99)	1.21 (1.05-1.41)	.009
55-64 (n = 498)	198 (15.05)	300 (15.72)	33.09 (28.48-37.7)	50.14 (44.47-55.82)	1.52 (1.27-1.81)	<.001
≥65 (n = 149)	61 (4.64)	88 (4.61)	8.13 (6.22-10.44)	11.72 (9.4-14.44)	1.44 (1.04-2.00)	.03
Residency						
Metropolitan (n = 2106)	866 (65.81)	1240 (64.96)	32.79 (30.61-34.97)	46.95 (44.34-49.57)	1.43 (1.31-1.56)	<.001
Nonmetropolitan (n = 1119)	450 (34.19)	669 (35.04)	24.63 (22.36-26.91)	36.62 (33.85-39.4)	1.49 (1.32-1.68)	<.001
Contributing drugs						
Opioid (n = 2632)	1032 (78.42)	1600 (83.81)	23.10 (21.73-24.55)	35.74 (34.03-37.53)	1.55 (1.43-1.67)	<.001
Heroin (n = 254)	144 (10.94)	110 (5.76)	3.22 (2.73-3.79)	2.46 (2.04-2.97)	0.76 (0.60-0.98)	.03
Fentanyl (n = 2108)	763 (57.98)	1345 (70.46)	17.08 (15.91-18.33)	30.10 (28.54-31.76)	1.76 (1.61-1.92)	<.001
Cocaine (n = 280)	116 (8.81)	164 (8.59)	2.60 (2.16-3.11)	3.67 (3.15-4.28)	1.41 (1.11-1.79)	.005
Cocaine and opioid (n = 223)	85 (6.46)	138 (7.23)	1.90 (1.54-2.35)	3.09 (2.61-3.65)	1.62 (1.24-2.12)	<.001
Psychostimulant (n = 1174)	465 (35.33)	709 (37.14)	10.41 (9.50-11.40)	15.84 (14.71-17.05)	1.52 (1.36-1.71)	<.001
Psychostimulant and opioid (n = 840)	308 (23.40)	532 (27.87)	6.89 (6.17-7.71)	11.88 (10.91-12.94)	1.72 (1.50-1.98)	<.001

Abbreviation: NA, not applicable.

^a^ Counts for year 2020 are provisional, likely incomplete, and subject to change.

^b^ A rate ratio of 1.45 is interpreted as 45% increase in the drug overdose death rate from 2019 to 2020.

^c^ Counts between 1 and 5 were suppressed according to the state data reporting policy. If the original size of the suppressed cell could be determined by subtraction from the total, then the exact number of the next smallest cell was also suppressed. Rates for fewer than 10 deaths were not calculated.

^d^ Includes residents with other race (eg, American Indian, Alaska Native, Asian, and Pacific Islander) and non-Hispanic ethnicity.

**Figure.**
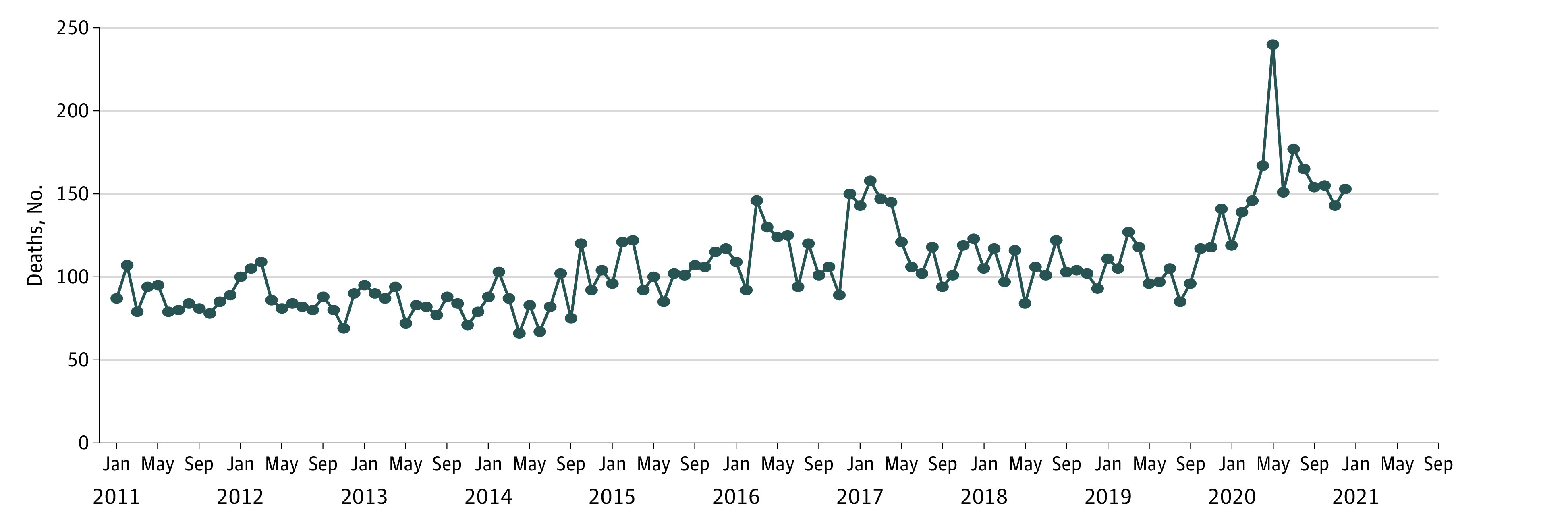
Kentucky Resident Drug Overdose Deaths, by Month, 2011-2020 Counts for year 2020 are provisional, likely incomplete, and subject to change.

Significant increases in mortality rates were observed for both sexes, Black non-Hispanic residents, White non-Hispanic residents, all age groups, and metropolitan and nonmetropolitan residency groups (Table). The highest 2020 age-specific rate was among residents aged 35 to 44 years (101.10 deaths per 100 000 population; 95% CI, 92.7-109.49 deaths per 100 000 population). Overdose risk increased by 57% (RR, 1.57; 95% CI, 1.23-2.00; *P* < .001) among Black non-Hispanic residents and by 45% (RR, 1.45; 95% CI, 1.34-1.56; *P* < .001) among White non-Hispanic residents. The relative rate increase was higher among male residents (RR, 1.58; 95% CI, 1.45-1.73) than female residents (RR, 1.22; 95% CI, 1.08-1.38), but was similar among metropolitan (RR, 1.43; 95% CI, 1.31-1.56) and nonmetropolitan (RR, 1.49; 95% CI, 1.32-1.68) residents.

There were increases in the rates of opioid-involved deaths (RR, 1.55; 95% CI, 1.43-1.67), especially fentanyl-involved deaths (RR, 1.76; 95% CI, 1.61-1.92), whereas the rate of heroin-involved deaths decreased (RR, 0.76; 95% CI, 0.60-0.98). There were significant increases in concomitant opioid involvement with cocaine (RR, 1.62; 95% CI, 1.24-2.12) and other psychostimulants (RR, 1.72; 95% CI, 1.50-1.98).

## Discussion

Our analysis indicated higher drug overdose mortality in 2020 compared with previous years,^6^ with a substantial increase early during the COVID-19 pandemic and sustained elevated counts through the end of 2020. Additional research on COVID-19–associated and non–COVID-19–associated factors is warranted. Significant increases in opioid involvement among cocaine-involved and other psychostimulant-involved deaths highlight the need for harm reduction outreach and access to naloxone among stimulant users. Fentanyl analog and novel synthetic opioid involvement is likely underreported because of limitations in postmortem toxicology to identify some of these substances and the lack of testing of all decedents with a designer opioid test panel. Significant increases in mortality across all demographic groups in Kentucky suggest an urgent need for a universal public health response.

## Notes

Supplement. **eAppendix.** Technical Notes on Methods eReferences
