# Supplementary material for: Trends in Drug Overdose Mortality Rates in Kentucky, 2019-2020
Source: JAMA Netw Open. 2021 Jul 13;4(7):e2116391. doi: 10.1001/jamanetworkopen.2021.16391 (PMC8278264; doi:10.1001/jamanetworkopen.2021.16391)
Supplement: Supplement. — eAppendix. Technical Notes on Methods eReferences [file jamanetwopen-e2116391-s001.pdf]

Supplemental Online Content

Slavova S, Quesinberry D, Hargrove S, et al. Trends in drug overdose mortality rates in Kentucky, 2019-2020. *JAMA Netw Open*. 2021;4(7):e2116391. doi:10.1001/jamanetworkopen.2021.16391

eAppendix. Technical Notes on Methods

eReferences

This supplemental material has been provided by the authors to give readers additional information about their work.

© 2021 Slavova S et al. *JAMA Network Open.*

eAppendix. Technical Notes on Methods

Data sources:

This study used electronic death certificate records from the Kentucky Office of Vital Statistics, Cabinet for Health and Family Services, supplemented with postmortem toxicology data from the Kentucky Drug Overdose Fatality Surveillance System[1] housed at the Kentucky Injury Prevention and Research Center, bona fide agent for the Kentucky Department for Public Health.

Study measures:

Drug overdose deaths were identified based on electronic death certificate records with an underlying cause-of-death coded with an ICD-10 (International Classification of Diseases, 10th Revision[2]) code in the range X40–X44 (unintentional drug poisoning), X60–X64 (intentional self-poisoning by drugs/suicide), X85 (homicide poisoning by drugs), or Y10–Y14 (poisoning by drug with undetermined intent).[3]

Drug overdose deaths with specific drug involvement: Multiple cause of death ICD-10 codes were used to identify the involvement of the following drugs/drug classes in the drug overdose deaths: T40.1 heroin; T40.0 – T40.4 or T40.6 opioids; T40.5 cocaine; T43.6 psychostimulants with abuse potential (e.g., methamphetamine, amphetamine, 3,4-Methylenedioxymethamphetamine (MDMA), methylphenidate; excluding cocaine).[4] Due to the lack of specific ICD-10 codes, involvement of fentanyl and fentanyl analogs was identified from narrative text analyses of the cause-of-death section on the death certificate, using an approach described by the Council of State and Territorial Epidemiologists[5] and a list of search terms for fentanyl and fentanyl analogs developed by the National Center for Health Statistics (NCHS)[6]. According to the NCHS, the monthly percentage of Kentucky drug overdose death certificates that did not list any specific drugs involved in an overdose death varied from 5.2% to 8.1% in the January 2019 – December 2020 period.[7] In such cases, the presence of any opioids, specific involvement of heroin or fentanyl and fentanyl analogs, cocaine, or other psychostimulants with abuse potential (specifically amphetamine or methamphetamine) was identified from the postmortem toxicology testing report.

The literal text in the cause-of-death section on the death certificates was queried for search terms (including drug names, misspellings, and metabolites) used previously by Hedegaard et al[6] (direct link to their table available from:

© 2021 Slavova S et al. *JAMA Network Open.*

https://ftp.cdc.gov/pub/Health_Statistics/NCHS/Publications/NVSR/68_12/Table.xlsx). The search terms were crosswalked to the following fentanyl and fentanyl analog principal variants:

Fentanyl and Fentanyl Analog Principal Variants

1	.BETA.-HYDROXYFENTANYL	21	DESPROPIONYL O-FLUOROFENTANYL
2	.BETA.-HYDROXYTHIOFENTANYL	22	FENTANYL
3	3-FLUOROISOBUTYRYLFENTANYL	23	FURANYLFENTANYL
4	3-METHYLFENTANYL	24	ISOBUTYRFENTANYL
5	4-FLUOROBUTYRFENTANYL	25	ISOBUTYRYLFENTANYL
6	4-FLUOROISOBUTYRLFENTANYL	26	METHOXYACETYLFENTANYL
7	4-FLUOROISOBUTYRYLFENTANYL	27	METHYLFENTANYL
8	4-METHOXYBUTYRFENTANYL	28	NORALFENTANIL
9	ACETYLFENTANYL	29	NORFENTANYL
10	ACRYLFENTANYL	30	O-METHYLACETYLFENTANYL
11	ACYLFENTANYL	31	OCFENTANIL
12	ALFENTANIL	32	ORTHO-FLUOROFENTANYL
13	BENZYLFENTANYL	33	P-FLUOROFENTANYL
14	BUTYRFENTANYL	34	P-METHOXYFENTANYL
15	CARFENTANIL	35	PARAFLUOROBUTYRFENTANYL
16	CROTONYL FENTANYL	36	PHENETHYLPIPERIDINE
17	CYCLOPROPYL FENTANYL	37	PHENYLFENTANYL
18	DEPROPIONYL FLUOROFENTANYL	38	REMIFENTANIL
19	DEPROPIONYLFENTANYL	39	SUFENTANIL
20	DESPROPIONYL FLUOROFENTANYL	40	TETRAHYDROFURAN FENTANYL
		41	VALERYLFENTANYL

Note: The search term 4-ANPP and its different spellings/misspelling are cross-walked to the principal variant DEPROPIONYLFENTANYL.

Denominators for crude rates: The denominator for the 2020 statewide rates was based on the U.S. Census Bureau's population estimate of the Kentucky resident population as of July 1, 2020.[8] The denominator for the 2019 statewide rates as well as the denominators for the 2019 and the 2020 group-specific rates were based on the 2019 bridged-race population estimates from the National Center for Health Statistics[9] because 2020 Kentucky resident population estimates by age group, race/ethnicity, gender, and county of residence were not available at the time the analysis was performed.

Analysis:

Rates, rate ratios, and 95% confidence intervals were modeled with Poisson distribution, using statistical software SAS 9.4 and proc GENMOD.[10] Rates based on small number of deaths should be interpreted

© 2021 Slavova S et al. *JAMA Network Open.*

with caution. Rates based on < 20 deaths are considered statistically unreliable. Data were suppressed when the number of deaths was ≤5 according to the Kentucky Department for Public Health data reporting policy. If the original size of the suppressed cell could be determined by subtraction from the total, then the exact number of the next smallest cell was also suppressed.

eReferences

1. Hargrove SL, Bunn TL, Slavova S, et al. Establishment of a comprehensive drug overdose fatality surveillance system in Kentucky to inform drug overdose prevention policies, interventions and best practices. *Injury prevention : journal of the International Society for Child and Adolescent Injury Prevention.* 2018;24(1):60-67.

2. WHO. International classification of diseases, tenth revision: version 2016. World Health Organization. 2016; http://apps.who.int/classifications/icd10/browse/2016/en.

3. Hedegaard H, Miniño AM, Warner M. Co-involvement of Opioids in Drug Overdose Deaths Involving Cocaine and Psychostimulants. *NCHS data brief.* 2021(406):1-8.

4. Hedegaard H, Miniño AM, Warner M. Drug Overdose Deaths in the United States, 1999-2019. *NCHS data brief.* 2020(394):1-8.

5. Davis J, Sabel J, Wright D, Slavova S. Epi tool to analyze overdose death data. 2015; http://www.cste.org/blogpost/1084057/211072/Epi-Tool-to-Analyze-Overdose-Death-Data.

6. Hedegaard H, Bastian BA, Trinidad JP, Spencer MR, Warner M. Regional Differences in the Drugs Most Frequently Involved in Drug Overdose Deaths: United States, 2017. *National vital statistics reports : from the Centers for Disease Control and Prevention, National Center for Health Statistics, National Vital Statistics System.* 2019;68(12):1-16.

7. NCHS. National Center for Health Statistics (NCHS) Vital Statistis Rapid Release. Table on Improvement of Data Quality, by State and Year. Available from https://www.cdc.gov/nchs/nvss/vsrr/vsrr_drug_datatable.csv. 2020.

8. U.S. Census Bureau. Evaluation Estimates. 2021; https://www.census.gov/programs-surveys/popest/technical-documentation/research/evaluation-estimates.html.

9. National Center for Health Statistics. Bridged-Race Resident Population Estimates, United States, State and County, for the years 1990 - 2019. 2021; https://wonder.cdc.gov/bridged-race-population.html.

10. SAS. Usage Note 24188: Modeling rates and estimating rates and rate ratios (with confidence intervals). 2021; https://support.sas.com/kb/24/188.html.

© 2021 Slavova S et al. *JAMA Network Open.*

© 2021 Slavova S et al. *JAMA Network Open.*
